# Healthcare provider perceptions and reported practices regarding opioid prescription for patients with chronic pain

**DOI:** 10.21203/rs.3.rs-3367358/v1

**Published:** 2023-09-27

**Authors:** Joseph Arthur, Tonya Edwards, Zhanni Lu, Doris Magdalene Amoateng, Kwame Koom-Dadzie, Hongxu Zhu, James Long, Kim-Anh Do, Eduardo Bruera

**Affiliations:** The University of Texas MD Anderson Cancer; The University of Texas MD Anderson Cancer; The University of Texas MD Anderson Cancer; The University of Texas MD Anderson Cancer; The University of Texas MD Anderson Cancer; The University of Texas MD Anderson Cancer; The University of Texas MD Anderson Cancer; The University of Texas MD Anderson Cancer; The University of Texas MD Anderson Cancer

**Keywords:** opioid, nonmedical, chronic cancer pain, prescribing

## Abstract

**PURPOSE:**

Data indicates that clinicians might be under-prescribing opioids for patients with chronic cancer pain, and this could impact adequate chronic pain management. Few studies have sought to understand healthcare provider (HCP) perceptions and practices regarding the prescription of opioids for chronic pain. We assessed HCP perceptions and practices regarding opioid prescription for patients with chronic pain since the onset of the COVID-19 pandemic.

**METHODS:**

An anonymous cross-sectional survey was conducted among 186 HCPs who attended an opioid educational event in April 2021 and 2022.

**RESULTS:**

61/143(44%) opioid prescribers reported reluctance to prescribe opioids for chronic pain. In a multivariate logistic model, younger participants (log OR −0.04, 95% CI: −0.085, −0.004; p = 0.033) and pain medicine clinicians (log OR −1.89, CI: −3.931, −0.286; p = 0.034) were less reluctant, whereas providers who worry about non-medical opioid use (NMOU) were more reluctant to prescribe opioids (log OR 1.58 95% CI: 0.77–2.43; p < 0.001). 53/143(37%) respondents had experienced increased challenges regarding opioid dispensing at pharmacies, and 84/179(47%) reported similar experience by their patients. 54/178(30%) HCPs were aware of opioid-related harmful incidents to patients or their families, including incidents attributed to opioid misuse by a household or family member.

**CONCLUSION:**

A significant number of opioid prescribers were reluctant to prescribe opioids for patients with chronic pain. Many reported challenges regarding dispensing of opioids at the pharmacies. These may be unintended consequences of policies to address the opioid crisis. Future measures should focus on addressing regulatory barriers without undermining the gains already made to combat the opioid crisis.

## INTRODUCTION

Prescription opioids remain the cornerstone of cancer pain management.[1] Regrettably, their nonmedical use has been implicated in the opioid overdose crisis which continues to be a major health care concern in the country. Over 40% of all opioid-related overdose deaths involved a prescription opioid alone.[2] Of the 3.8 million Americans who misuse prescription opioids, approximately 50% obtain the medication from a friend or relative.[3] Moreover, prescription opioids have been reported as gateway drugs among many heroin users.[4] Data suggests that the opioid crisis worsened after the onset of the COVID-19 pandemic. [5–7] All of these facts, coupled with the increased rules, regulations, and policies to combat the opioid crisis might be negatively impacting legitimate opioid access for patients who genuinely need the medication to treat intractable cancer pain. Data indicates that clinicians are under-prescribing opioids for patients with cancer,[8, 9] and this might be resulting in an inadvertent undertreatment of cancer pain.[10–12]

A careful balance of the judicious use of opioids with their risk for non-medical use among patients with chronic pain is therefore critically needed. It starts by gaining a broad understanding of provider perceptions and practices related to opioid prescriptions, especially after the onset of the COVID-19 pandemic. However, this has not been sufficiently examined in literature. The main objective of our study was to assess the reluctance among healthcare providers to prescribe opioids to patients with chronic pain since the onset of the COVID-19 pandemic. We also assessed other opioid prescribing practices and perceptions among healthcare providers.

## METHODS

### Survey design

This was an anonymous cross-sectional survey conducted among healthcare providers who attended the Interdisciplinary Pain and Opioid Crisis Seminar, a one-and-a-half-day educational event organized annually by the Department of Palliative, Rehabilitation and Integrative Medicine at the University of Texas MD Anderson Cancer Center in April 2021 and 2022. Event attendees were health care providers who prescribed opioids (medical oncologists, pain and palliative care physicians, emergency room physicians, primary care physicians, advanced practice providers), administered opioids (nurses, pharmacists), or were involved with patients on opioids (psychiatrists, psychologists, social workers, case managers, and clinical leaders).

### Participants and study procedure

Eligible participants were seminar attendees who could understand, read, write, or speak English, and were willing to participate in the survey. Participants received an email on the eve of the event asking them to complete an anonymous web-based survey. Those who were unable to complete the survey received daily email reminders up to 3 days after the end of the meeting. Participants were informed that survey participation was voluntary. The web-based survey was developed using Qualtrics and consisted of a questionnaire developed by the study investigators.

### Survey Instrument

The **Opioid Prescribing Survey (APPENDIX A)** was developed by study investigators (JA, EB) after a thorough review of previous studies that assessed healthcare providers’ beliefs and attitudes towards opioid prescribing for patients with chronic pain.[13–15] This pilot survey was reviewed for its content by other members of the event organizing committee who were considered content experts, and it was further revised for clarity and face validity after a blueprint meeting including research associates and biostatisticians. The survey consists of 26 questions on participants’ perceptions and experiences with patients receiving prescription opioids for chronic pain since the onset of the COVID-19 pandemic. Participants who prescribed opioids for chronic pain answered specific questions related to their prescribing practices. Survey responses were mostly based on a 5-point Likert scale ranging from “strongly disagree” to “strongly agree”, with a few open-ended questions. Most of the question items were adapted from previously tested instruments such as the Concerns About Analgesic Prescriptions Questionnaire[14, 15] and the Opioid Therapy Survey.[13] The survey also included questions on participants’ demographic and clinical characteristics such as age, gender, race, profession, and duration of practice. The whole survey had an estimated completion time of 10 minutes.

### Statistical Analysis

We calculated the percentage of responders who selected ‘agree’ or ‘strongly agree’ (for each individual survey question) along with a 95% confidence interval. With an estimated 200 responses to each question, the confidence intervals were expected to be no wider than 14% (4 * sqrt (0.5*0.5/200)). Descriptive statistics such as frequency and percentage for categorical data, and median with interquartile range (IQR) for continuous variables, were used to summarize the participants’ survey responses. Chi-squared test was used to assess the association between categorical variables. Logistic regression was fit using participant characteristics (age, gender, race, level of education, as age, sex, race, type of profession, years of experience, specialty, worry about NMOU in their patients, worry about opioid-related harm) as independent variables and the primary outcome of interest (providers’ reluctance to prescribe opioids) as the dependent variable. Independent variables with coefficient q-value (local FDR) less than 0.1 were considered of interest. P-values less than 0.05 were considered statistically significant. The data were analyzed with R version 4.1.1.

## RESULTS

Overall, 207 of 387 (54%) survey recipients completed the survey. Twenty-one non-US respondents were excluded from the analysis because they were from countries with limited experience and exposure to issues related to prescription opioids and were likely to have varied views that might not be applicable in the US. One hundred and eighty-six participants were therefore included in the analysis. Participants’ demographic and clinical characteristics are summarized in Table 1. Median (IQR) age of participants was 46 (39–56) years. Majority were female (127 [69%]), White (68[38%]), advanced practice providers (103 [55%]) and had over 10 years of clinical experience (88 [51%]).

Table 2 summarizes participants’ perceptions, experiences, and opioid prescribing practices for patients with chronic pain. The vast majority (151[84%]) were more concerned about the mental health of patients with chronic pain since the onset of the COVID-19 pandemic. Only 84 participants (48%) reported feeling confident in caring for patients with NMOU. 54 participants (30%) were aware of incidents of harm to their patients or their families due to prescription opioids. Although majority of opioid prescribers felt comfortable prescribing opioids (86[61%]), over half of them (76 [54%]) were increasingly worried about NMOU in their patients and a considerable number had been reluctant to prescribe opioids for chronic pain (61[44%]). Only a few of them reported ultimately prescribing opioids less frequently (22[15%]). Almost half of all respondents (84[47%]) reported their patients had experienced increased difficulty filling their opioid prescriptions, and over a third of opioid prescribers (53[37%]) had experienced difficulties themselves in working with pharmacies when prescribing opioids.

**Figure 1:**
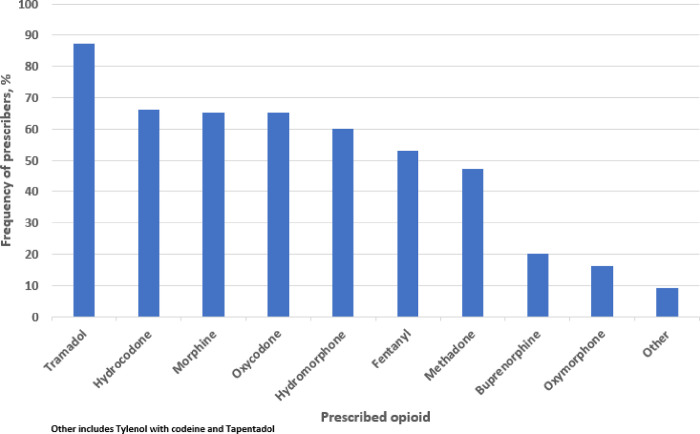
Distribution of prescribers of different opioids The figure shows the percentage of prescribers who prescribed different opioids.

**Figure 2:**
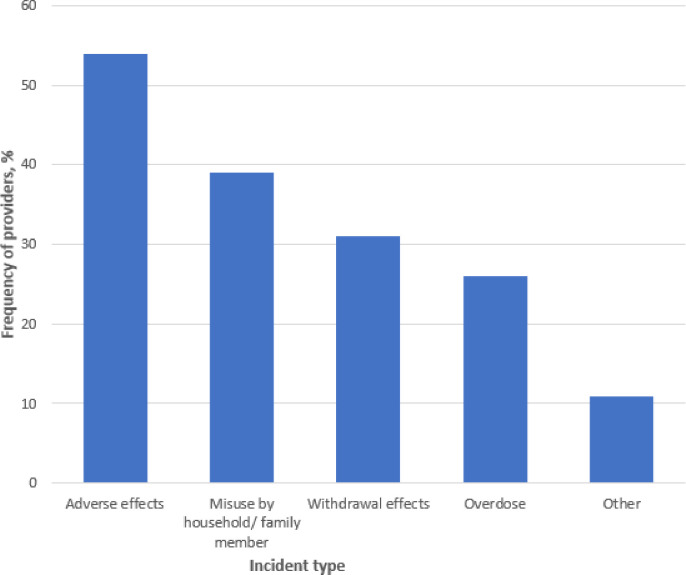
Percentage of providers reporting different prescription opioid-related harmful incidents in their patients The figure illustrates the proportion of providers who reported being aware of different prescription opioid-related harmful incidents in their patients.

Akaike Information Criteria (AIC) was used to select the best multivariate logistic regression model to associate provider demographic variables and prompt responses with reluctance to prescribe opioids. Table 3 provides information on the factors associated with providers’ reluctance to prescribe opioids in the best fitting model. Younger age (log OR −0.04, 95% CI: −0.085, −0.004; p = 0.033) and pain medicine physicians as compared to oncologists (log OR −1.89, CI: −3.931, −0.286; p = 0.034) were less reluctant to prescribe opioids. Providers who worry about NMOU were more reluctant to prescribe opioids as compared to those who don’t (log OR 1.58 95% CI: 0.770, 2.433; p < 0.001). Figure 1 shows the distribution of participants who prescribed certain common opioids. Most participant prescribers reported Tramadol (105/124[87%]), followed by hydrocodone (82/124[66%]). Acetaminophen with Codeine and Tapentadol (11/124[9%]) were the least prescribed opioids. Figure 2 illustrates the proportion of providers who reported being aware of prescription opioid-related harmful incidents in their patients. Fifty four percent (89/165) reported incidents due to opioid-related adverse effects. Almost 40% (65/165) of reported incidents were attributed to opioid misuse by a household or family member.

## DISCUSSION

In this study, a significant number of opioid prescribers felt comfortable prescribing opioids but were increasingly worried about NMOU in their patients, and many felt reluctant to prescribe opioids for chronic pain since the onset of COVID-19 pandemic. Healthcare providers are understandably cautious about prescribing opioids for their patients due to several reasons. Prescription opioids are noted to play a major role in the opioid crisis, which was exacerbated by the COVID-19 pandemic.[5, 6, 16] Recent data indicates that 24% of all drug overdose deaths from 2020 to 2021 were related to prescription opioids.[17] Non-medical use of prescription opioids may inadvertently result from relaxed provider prescribing practices.[18, 19] Moreover, the medical community is apprehensive about opioid prescriptions[20] because some members of the general public hold physicians accountable for the opioid crisis.[21] Some of the increased rules, regulations, and policies in response to the opioid crisis may have also become impediments to prescribing opioids and are potentially obstructing patients’ legitimate access to opioids for the treatment of severe cancer pain,[10, 12, 22–24] especially among the disadvantaged racial minorities.[25] Fortunately, only a few of the prescribers in this survey reported that they did prescribe opioids less frequently.

One strategy to increase provider confidence and willingness to prescribe opioids for patients with chronic pain is to increase interdisciplinary clinical support for prescribers. Studies have shown that clinicians perceive caring for patients with NMOU behavior as stressful and time consuming.[13, 26] This, coupled with the numerous systemic barriers they face when prescribing opioids, and the limited time availability in busy clinical settings, serve as disincentives to opioid prescribing. Clinical teams should therefore establish programs that provide access to services and resources for NMOU management and offer an extra layer of support for providers.[27] This may help reduce burnout among already heavily burdened clinical teams. Emerging opioid stewardship programs such as the Compassionate High Alert Team (CHAT) model provide comprehensive interdisciplinary care to patients with NMOU behaviors and have been shown to significantly reduce problematic opioid use.[28]

Participants were generally more concerned about their patients’ mental health since the onset of the COVID-19 pandemic. Theis is probably because the pandemic led to increased rates of depression and anxiety which are common co-occurring conditions among patients at risk for opioid use disorder.[29, 30] Data indicates that the opioid crisis worsened after the onset of the COVID-19 pandemic.[5, 6, 16] Urine drug test positivity rates in individuals with a diagnosis of, or at risk of substance use disorders increased significantly during the post-pandemic period.[5–7] One plausible reason for this observation is the impact of psychosocial stressors related to the COVID-19 pandemic[7] especially among individuals who had pre-existing sources of stress such as the diagnosis of cancer. Cancer patients already face significantly stressful conditions from disease burden and complications from their treatment.

A significant number of survey respondents recounted their patients’ increased difficulties with filling their opioid prescriptions as well as their own challenges working with pharmacies to dispense opioid prescriptions. This may be another undesirable consequence of measures implemented to address the opioid crisis. Recently, there has been an increase in the number of state and insurance[31] limitations on opioid dispensing. Medicare Prescription Drug Plan coverage for opioids commonly used to treat chronic cancer pain has become increasingly restrictive.[32] State and pharmacy mandated opioid dose and duration limits have become more prevalent and these unfortunately affect patients with chronic cancer-related pain.[24, 33, 34] Some pharmacies are required to follow stringent measures before dispensing opioids. These measures result in avoidable time demands on prescribers and pharmacists as well as undue stress and potential medical harm to patients.[35] Pharmacists acknowledge uncertainties regarding the extent to which they should operate without being an impediment to patients’ care.[36] It is important to recognize that they also face unique challenges as they serve as safeguards against illegitimate opioid access while ensuring efficient care delivery to patients with chronic pain. The relevant stakeholders should therefore find ways to address these regulatory issues which have become major barriers to effective pain control in patients with chronic pain.

Tramadol was the most prescribed opioid among survey respondents. This is consistent with prior research which found that tramadol is increasingly being prescribed for patients with chronic cancer pain after hydrocodone was reclassified as a scheduled II opioid in October 2014.[9] Perhaps, its relatively lax prescribing requirements and regulatory scrutiny make tramadol an appealing and popular opioid among some clinicians. Regrettably, tramadol is a weak mu opioid receptor agonist which can lower threshold for seizure activity[37] and it may cause serotonin syndrome by interacting with drugs that are frequently used in patients with cancer such as antiemetics, antidepressants, and neuroleptics.[38] It’s frequent use can result in increased incidence of suboptimal pain control even in patients with mild to moderate pain. A multicenter randomized control trial comparing the efficacy of weak opioids such as tramadol with low dose morphine in patients with moderate pain found that tramadol was significantly less effective than low dose morphine.[39]

A significant number of participants reported opioid-related harmful incidents in their patients that were attributed to opioid misuse by a household or family member. This important finding illustrates the fact that the risk related to NMOU transcends the individual to affect those around them. Clinicians should therefore be aware of the potential broader repercussions with unsafe prescribing practices. Family members and caregivers caring for patients with chronic cancer pain may have increased access and exposure to substantial amounts of opioids. Over half of the 3.8 million Americans who misuse prescription opioids obtain the medication from a friend or relative.[3] In another survey, greater than 50% of Americans indicated that they knew someone close to them with an opioid use disorder.[40] This further underscores the need for providers to intensify patient education on safe and rational use, storage, and disposal of opioids.[41, 42]

One limitation of our study is that participants were recruited from a single opioid educational event. The findings may therefore not be generalizable to other participants in different settings. Second, details of the reasons why participants were reluctant to prescribe opioids was not captured in real time. Future studies should aim at specifically obtaining such important information directly from participants since this will be critical in increasing understanding of provider challenges with opioid prescriptions. Third, the study findings were based on participant self-report which might not necessarily represent the actual opioid prescribing practices of the study participants.

## CONCLUSION

Healthcare providers were frequently comfortable prescribing opioids for chronic pain but reluctant to do so. Many reported frequent challenges that they and their patients faced when filling opioid prescriptions at the pharmacies. Tramadol, a weak opioid with notable side effect profile, was the most prescribed opioid among prescribing participants. These findings might be unintended consequences of policies implemented to combat the opioid crisis. Future studies should therefore focus on strategies to reduce barriers to effective opioid pain management without undermining the progress that has been made thus far to mitigate NMOU and combat the opioid overdose crisis.

## Figures and Tables

**Table 1:** Demographic and clinical characteristics of study participants (N = 186)

Covariate	Level	Participants, n (%)^a^
**Age, years, median (IQR)**		46 (39, 56)
**Gender**	Female	127 (69)
	Male	56 (30)
	Other	2 (1)
**Race/ ethnicity**	White	68 (38)
	Asian	67 (37)
	Black or African American	15 (8)
	Hispanic/Latino	13 (7)
	Other	18 (10)
**Profession**	Physician	57 (31)
	Registered Nurse	21 (11)
	Advanced practice provider	103 (55)
	Other	5 (2.6)
**Years of experience**	< 1 year	9 (6)
	1–10 years	74 43)
	> 10 years	88 (51)
**Specialty**	Oncology	53 (29)
	Palliative Medicine	51 (27)
	Pain Medicine	18 (10)
	General Practice	13 (7)
	Surgery	13 (7)
	Anesthesiology	6 (3)
	Other	31 (17)

a Some covariates had missing data.

Abbreviations: IQR, interquartile range

**Table 2:** Participants' perceptions, experiences, and opioid prescribing practices for patients with chronic pain since the onset of COVID-19 pandemic

Question	N	# Agree n (%)	95% Confidence Interval
I have prescribed opioids less frequently.	143	22 (15)	10, 23
I have increasingly prescribed non-narcotic analgesics rather than opioids for pain control.	141	53 (38)	30, 46
I have been reluctant to prescribe opioids for chronic pain.	140	61 (44)	35, 52
I am increasingly worried about nonmedical opioid use in my patients.	142	76 (54)	45, 62
I am more worried about harm to my patients when prescribing opioids.	139	72 (52)	43, 60
I find patients with pain more stressful to deal with.	142	69 (49)	40, 57
I have decreased in confidence to prescribe opioids.	143	23 (16)	11, 23
I feel comfortable prescribing opioids.	140	86 (61)	53, 69
Prescribing opioids is more stressful due to the pandemic.	143	37 (26)	19, 34
I have had more difficulties working with pharmacies when prescribing opioids.	143	53 (37)	29, 46
Treating patients with pain has increasingly been a problem	177	74 (42)	35, 49
I feel patients have increasingly engaged in non-medical opioid use.	178	58 (33)	26, 40
I have been aware of more incidents of harm (to patients or their families) due to prescription opioids.	178	54 (30)	24, 38
I am more concerned for my patients' mental health.	179	151 (84)	78, 89
I feel confident in caring for patients with nonmedical use issues.	176	84 (48)	40, 55
My patients are reporting increased difficulty filling their opioid prescriptions.	179	84 (47)	39, 55

**Table 3:** Multivariable logistic regression model of factors associated with providers' reluctance to prescribe opioids*

Factor	Log Odds Ratio (95% Confidence interval)	P value
Age	−0.04 (−0.085, −0.004)	0.033
Worry about nonmedical opioid use	1.58 (0.770, 2.433)	<0.001
**Specialty**		
Anesthesiology	0.24 (−2.185, 2.727)	0.841
General Practice	−0.22 (−1.738, 1.259)	0.773
Other	−0.67 (−2.031, 0.641)	0.322
Pain Medicine	−1.89 (−3.931, −0.286)	0.034
Palliative Medicine	0.65 (−0.416, 1.758)	0.235
Surgery	−0.45 (−2.650, 1.504)	0.660
Oncology	1 (ref)	

* Positive log odds ratios indicate greater reluctance to prescribe opioids.

## Data Availability

Data is stored and can be accessed from password-protected institution computers at MD Anderson Cancer Center, behind the institution firewall.
